# Pan-immune-inflammation value independently predicts disease recurrence in patients with Merkel cell carcinoma

**DOI:** 10.1007/s00432-022-03929-y

**Published:** 2022-01-31

**Authors:** T. Gambichler, S. Said, N. Abu Rached, C. H. Scheel, L. Susok, R. Stranzenbach, J. C. Becker

**Affiliations:** 1 Skin Cancer Center, Department of Dermatology, Ruhr-University Bochum, Gudrunstraße 56, 44791 Bochum, Germany; 2 Translational Skin Cancer Research, DKTK Partner Site Essen/Düsseldorf, West German Cancer Center, Dermatology, University Duisburg-Essen, Essen, Germany; 3 German Cancer Research Center (DKFZ), Heidelberg, Germany

**Keywords:** Pan-immune-inflammation value, Neutrophil-to-lymphocyte ratio, Platelet-to-lymphocyte ratio, Merkel cell carcinoma, Biomarker

## Abstract

**Purpose:**

We aimed to determine whether the pan-immune-inflammation value (PIV) of patients with Merkel cell carcinoma (MCC) at primary diagnosis differs from controls and whether it is associated with disease stage and outcome.

**Methods:**

In this retrospective study, we recruited MCC patients with stage I–III. PIV was calculated from absolute complete blood cell counts obtained within one week at MCC diagnosis as follows: [neutrophils (10^3^/mm^3^) × platelets (10^3^/mm^3^) × monocytes (10^3^/mm^3^)]/lymphocytes (10^3^/mm^3^). As controls, we studied age–gender-matched cutaneous melanoma (CM, stage I–III) patients and healthy controls (HC). Univariate and multivariate statistics were used.

**Results:**

The median PIV in MCC patients was significantly increased compared to both CM patients as well as healthy controls. PIV of MCC patients in stage II and III was significantly higher compared to stage I patients. ROC analysis revealed that MCC recurrence was significantly associated with a PIV greater than 372 [*p* < 0.0001, Youden index 0.58; hazard ratio: 4 (95% confidence interval: 1.7 to 9.2)]. In multivariate analysis, only a PIV greater than 372 and higher MCC stage were determined as independent predictors for disease recurrence.

**Conclusion:**

We determined, for the first time, the prognostic ability of the promising blood-based biomarker PIV in MCC patients and observed that PIV is increased in MCC patients in dependence on disease stage and independently predicts MCC recurrence.

## Introduction

Merkel cell carcinoma (MCC) is a highly aggressive neoplasm of the skin. Major risk factors for MCC development are sun exposure, old age, and immunosuppression. The Merkel cell polyomavirus (MCPyV) is clonally integrated into the majority of MCCs in the Northern hemisphere. By contrast, MCPyV-negative MCC is associated with chronic ultraviolet (UV) exposure and harbors multiple UV-associated DNA mutations (Becker et al. 2017, 2019). In many malignancies, systemic inflammatory responses can be significant determinants of disease progression and survival. As a consequence, several immune-based prognostic scores have been investigated as predictors of prognosis and treatment response in several malignancies including skin cancers such as cutaneous melanoma (CM). These scores include neutrophil-to-lymphocyte ratio (NLR), platelet-to-lymphocyte ratio (PLR), and monocyte-to-lymphocyte ratio (MLR) (Zaragoza et al. 2016a; Zaragoza et al. 2016b; Robinson et al. 2020; Hernando-Calvo et al. 2021; Ludwig et al. 2021; Sahin et al. 2021). Recently, a more complex complete blood count (CBC)-based biomarker of systemic inflammation, the pan-immune-inflammation value (PIV), has been developed, but a systematic assessment of its usefulness as a biomarker of prognosis or treatment response in skin cancers has not yet been (Fucà et al. 2020; Hernando-Calvo et al. 2021; Susok et al. 2022). The PIV includes four CBC-based parameters: neutrophil, platelet, monocyte, and lymphocyte counts. In several studies including malignancies, such as metastatic CM, colorectal carcinoma and non-small cell lung carcinoma, PIV was independently associated with overall survival and progression-free survival (Fucà et al. 2020; Corti et al. 2021; Fuca et al. 2021; Zeng et al. 2021). Although biomarkers of systemic inflammation including NLR and PLR, have been investigated in patients with MCC, PIV has not yet been studied in this rare malignancy (Zaragoza et al. 2016b; Garnier et al. 2018; Naseri et al. 2020; Nghiem et al. 2021). Here, we aimed to determine whether PIV of patients with MCC at primary diagnosis differs from controls and whether it is associated with disease stage and outcome.

## Materials and methods

### MCC patients

In this retrospective study, we recruited MCC patients with stage I–III who were managed at the Skin Cancer Center of the Ruhr-University Bochum also including a CBC count performed at primary diagnosis. This patient group was compared to both an age- and gender-matched healthy control group as well as an age- and gender-matched group of patients with cutaneous melanoma (stage I–III). MCC restaging was performed in accordance with the eighth edition of the AJCC guidelines (Becker et al. 2019). This study was approved by the local ethics review board of the Medical Faculty of the Ruhr-University Bochum (#16-5985). Patients were treated in accordance with the German guidelines for MCC [9]. Accordingly, following primary excision with a 1–2 cm safety margin and sentinel lymph node biopsy, patients received adjuvant local radiotherapy for the tumor bed and draining lymph node basin. Metastatic lymph node disease was treated with complete lymphadenectomy. Patients with un-resectable stage III MCC received radiotherapy, electro-chemotherapy, and/or systemic chemotherapy or immune checkpoint inhibitors (Becker et al. 2019).

### Laboratory parameters

PIV was calculated from absolute CBC counts obtained within one week at MCC diagnosis as follows: [neutrophils (10^3^/mm^3^) × platelets (10^3^/mm^3^) × monocytes (10^3^/mm^3^)]/lymphocytes (10^3^/mm^3^). At the same time, lactate dehydrogenase (LDH) and C-reactive protein (CRP) were also determined (Fucà et al. 2020; Fuca et al. 2021).

### Statistical analyses

Data analysis was performed using the statistical package MedCalc Software version 19.1.7 (MedCalc Software, Ostend, Belgium). Distribution of data was assessed by the D ‘Agostino–Pearson test. Receiver operating characteristics (ROC) analyses, including the area under curve (AUC) and the Youden index, were performed to determine optimal cut-off values using continuous PIV values and the dichotomous classification variables “MCC recurrence” and “MCC-specific death” (Schistermann et al. 2008; Polley and Dignam 2021). Univariate analysis was performed using the Mann–Whitney test, Kruskal–Wallis ANOVA, Spearman’s correlation procedure, and Chi^2^ test. Survival analysis was performed using the Kaplan–Meier method and a multivariate approach by means of Cox proportional hazards regression, in which we included all parameters with *p* values of 0.1 and smaller on the basis of univariate analysis. *p* values < 0.05 were considered significant.

## Results

Forty-nine patients [median age: 77 years (51–95); 25 males, 24 females)] were studied, including 40 (81.6%) patients with Merkel cell polyomavirus (MCPyV)-positive MCC. Available clinical data for these patients are shown in Table. 1. This patient group was compared to a healthy control group [*n* = 42; median age: 74 years (37–96); 22 males, 20 females] as well as a group of patients with cutaneous melanoma [stage I–III at first diagnosis; median age: 74 (50–93); 37 males, 34 females]. Importantly, median age and gender of MCC patients did not significantly differ from melanoma and healthy controls (*p* = 0.13 and *p* = 0.18 for age, *p* = 0.72 and *p* = 0.54 for gender, respectively). Moreover, the distribution of disease stages did not differ between MCC patients and melanoma controls (*p* = 0.69). Strikingly, the median PIV in MCC patients was significantly increased compared to both CM patients as well as healthy controls [*p* = 0.0002, MCC PIV = 507 (31–2648), CM PIV = 333 (33–2473), healthy PIV = 206 (52–1638), Fig. 1]. Additionally, as shown in Fig. 2, the PIV of MCC patients in stage II and III was significantly higher compared to stage I patients (*p* = 0.026). As expected for a marker of inflammation, the PIV of MCC patients significantly correlated with CRP levels (*r* = 0.44; *p* = 0.0017), but surprisingly not with the age of MCC patients (*p* = 0.15).

**Table 1:** Clinical characteristics of patients with Merkel cell carcinoma (MCC, *n* = 49), including data of the pan-immune-inflammation value (PIV) for MCC patients and controls

Parameters	Data
Age at diagnosis	77 years (51–95)
Sex	
M/f	25 (51%)/24 (49%)
Primary MCC	
High-risk localization: head/neck (no/yes)	30/19 (61.2/38.8%)
MCPyV (negative/positive)	9/40 (18.4/81.6%)
Immunosuppression	
No/yes	39 (79.6%)/10 (20.4%)
Tumor stage at diagnosis and median (range) PIV	
(AJCC 2018)	I 21 (42.9%)–314 (31–1551)
	II 17 (34.7%)–507 (226–2648)
	III 11 (12.4%)–787 (150–1680)
*p* value	= 0.027 (stage II and III vs. stage I)
Outcome	
5 year MCC recurrence (no/yes)	26 (53.1%)/23 (46.9%)
Median time to recurrence (months)	14 (IQR: 6–59)
5-year MCC (survived/deceased)	35 (63.6)/20 (36.4%)
Median time to death (months)	28 (IQR: 11.8–60)
Median (range) PIV	
MCC patients	507 (31–2648)
Melanoma controls	333 (38–2473)
Healthy controls	206 (52–1638)
*p* value	= 0.0002
ROC analyses	
PIV	
MCC recurrence*	AUC: 0.79 (95% CI 0.65–0.89); *p* < 0.0001; *J* = 0.58; criterion: > 372
MCC death*	AUC: 0.67 (95% CI 0.52–0.8; *p* = 0.024; *J* = 0.44; criterion: > 412

Data of ROC curve analyses are also provided

*MCPyV* Merkel cell polyomavirus; *J*  Youden index

*Classification variables

**Fig. 1:**
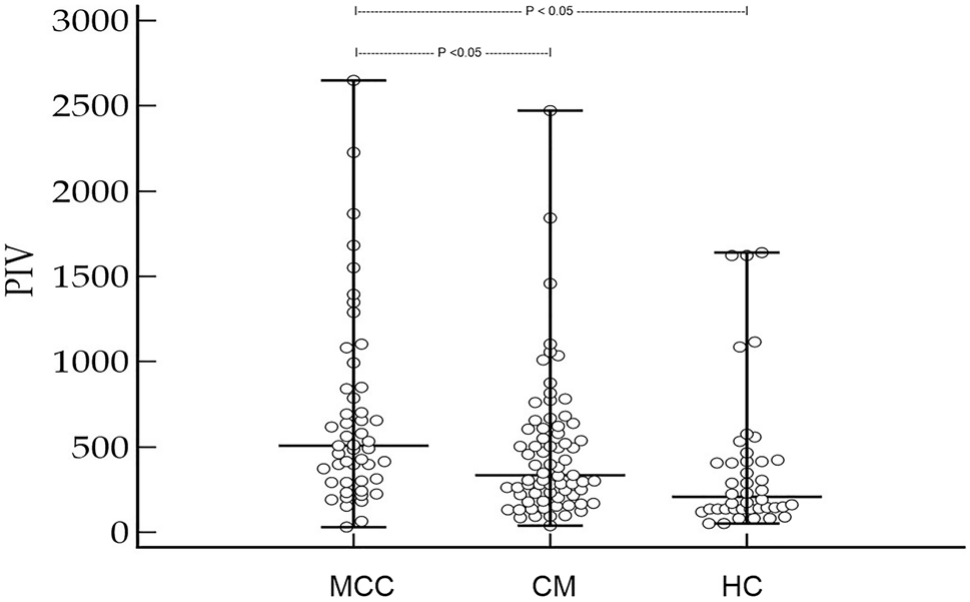
Merkel cell carcinoma (MCC) patients in stage I–III have an significantly increased pan-immune-inflammation value (PIV) when compared to patients with cutaneous melanoma (CM) in stage I–III and healthy controls (HC, Kruskal–Wallis ANOVA *p* = 0.0002)

**Fig. 2:**
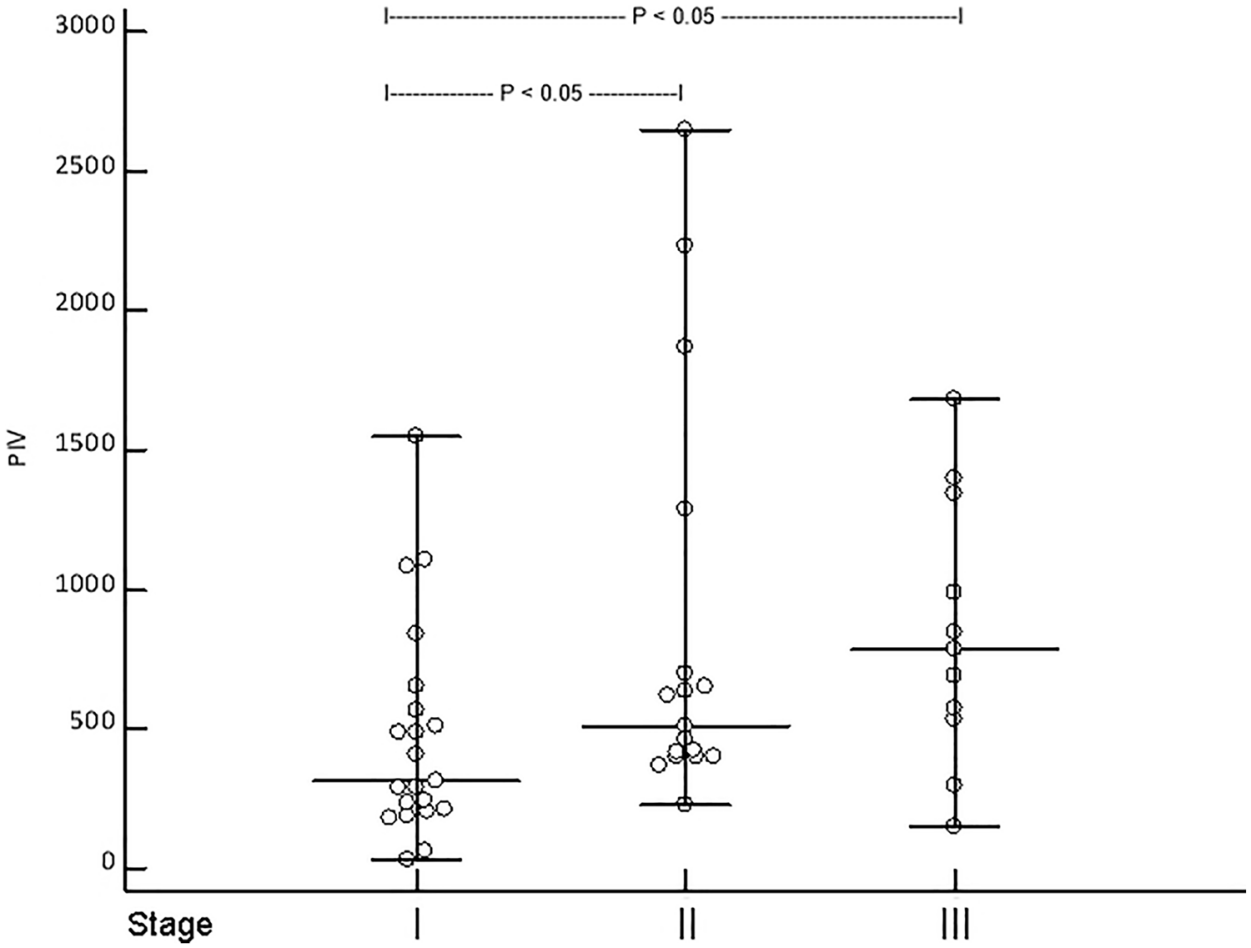
Showing the pan-immune-inflammation value (PIV) of patients (*n* = 49) with Merkel cell carcinoma (MCC) in stage I [median (range) PIV: 314 (31–1551)], II [median (range) PIV: 507 (226–2648)], and III median (range) PIV: 787 (150–1680)] at first diagnosis (**a**). Kruskal–Wallis ANOVA revealed that patients in stage II and III had a significantly higher PIV than patients in stage I (*p* = 0.026)

**Fig. 3:**
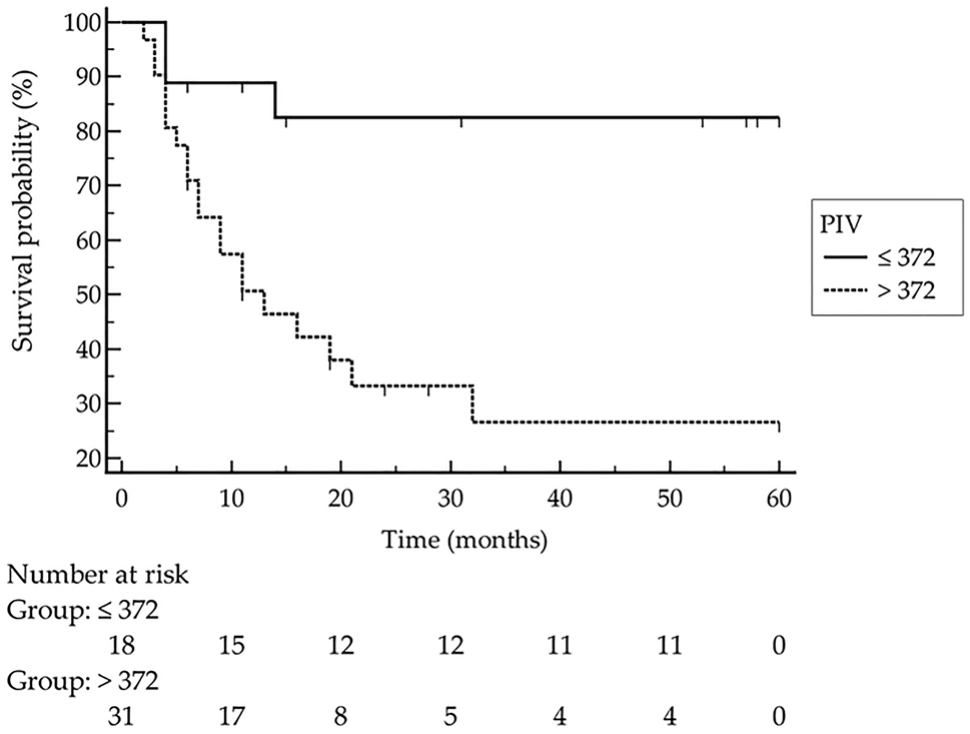
Kaplan–Meier curve clearly demonstrates that patients with a pan-immune-inflammation value (PIV) > 372 had significantly worse outcome with respect to MCC recurrence [*p* = 0.0015; hazard ratio: 4 (95% confidence interval: 1.7–9.2)]

ROC analysis revealed that MCC recurrence was significantly associated with a PIV greater than 372 (*p* < 0.0001, Youden index 0.58). Indeed, logistic regression curves showed that patients with PIV greater than 372 had significantly higher recurrence rates than patients with a PIV below 372 [Fig. 3, *p* = 0.0015, hazard ratio: 4 (95% confidence interval: 1.7–9.2)]. As expected, univariate analysis confirmed that higher MCC stage was significantly associated with disease recurrence (*p* = 0.0004). Taking into account different clinical parameters (age, sex, immunosuppression, MCPyV status, LDH, CRP, PIV class, disease stage), age above 75 years, PIV greater than 372 and higher stage of disease were all significantly associated with death (*p* = 0.011, *p* = 0.041 and *p* = 0.049, respectively). However, in multivariate analysis (including PIV class, increased CRP, and MCC stage), only a PIV greater than 372 and higher MCC stage were determined as independent predictors for disease recurrence (*p* = 0.014, *p* = 0.028, respectively). With respect to MCC-specific death, multivariate analysis (including age over 75 years, PIV class, disease stage, and elevated CRP) revealed that age over 75 years was the only significant independent predictor for worse survival (*p* = 0.0068).

## Discussion

The investigation of potential biomarkers in patients with MCC is crucial for patient stratification in the era of increasingly for personalized treatment strategies- For example, with respect to adjuvant treatment choices and the frequency of follow-up, biomarkers that accurately predict recurrence, are highly desirable. A wealth of research indicates that systemic inflammation plays a crucial role in tumor development, progression, and metastasis (Liu et al., 2015). On the one hand, pro-tumorigenic cytokines secreted by neutrophil granulocytes and thrombocytes, including vascular endothelial growth factor, tumor necrosis factor-α, and interleukin-10, have been suggested to contribute to tumor progression. On the other hand, monocytes as well as lymphocytes can exert antitumoral effects by increasing immune responses against cancer cells (Mirili et al. 2019). Systemic immune-inflammation prognosis scores, including NLR and PLR, have previously been reported to be of prognostic value in many different types of malignancy (Templeton et al. 2014; Liu et al. 2015; Nishijima et al. 2015; Zaragoza et al. 2016; Zhong et al. 2017; Mirili et al. 2019; Robinson et al. 2020; Hernando-Calvo et al. 2021; Ludwig et al. 2021). Thus, NLR and PLR belong to the most intensely investigated immune-inflammatory blood-based markers in malignancies including, in particular, melanoma, and are also associated with the clinical outcomes of patients treated with immunotherapy and targeted therapy (Zaragoza et al. 2016; Robinson et al. 2020; Hernando-Calvo et al. 2021; Ludwig et al. 2021; Sahin et al. 2021). However, given the complexity of the inflammation network, other cellular components not assessed by NLR and PLR may also have an influence on cancer progression and response to therapy. By capturing changes in four different cell types, the PIV provides a broad assessment of inflammation that might be particularly relevant with respect to potential pro-tumorigenic effects. In support of this consideration, a recent retrospective analysis of metastatic melanoma patients treated first-line with immune checkpoint inhibitors (ICI) or targeted therapy, demonstrated that a high baseline PIV was independently associated with poor progression-free and overall survival (Fucà et al. 2021). Moreover, a high PIV was also associated with primary resistance to both ICI and targeted therapy (Fucà et al. 2021). Another novel CBC-based biomarker represents the systemic immune-inflammation index (SII = platelets × neutrophils/lymphocytes) being a promising prognostic tool in cancers of the liver, pancreas, lungs, and gut (Zhong et al. 2017). Furthermore, Ludwig et al. (2021) assessed the SII in uveal melanoma and observed that low baseline SII was an independent predictor for prolonged overall survival (Ludwig et al. 2021).

Apart from CBC-based markers, many other biomarkers have previously been investigated. Despite routine use by many clinicians, neuron-specific enolase and chromogranin A blood levels do not correlate with MCC recurrence and MCC-specific survival (Gaiser et al. 2015). Similar to the present data, Donizy et al. (2021) showed on multivariate analysis that only ulceration and age were independent predictors of worse survival. However, they also demonstrated that tumoral PD-L1 expression and increased density of intra-tumoral CD8 + cells and FOXP3 + lymphocytes seem to be potential prognostic predictors in a subset of MCC patients (Donizy et al. 2021). Moreover, Harms et al. (2021) recently found that MCPyV-positive MCC patients also showing decreased tumoral expression of granzyme B and IDO-1 have shorter survival when compared to MCPyV-negative MCC patients. Other reports suggested that PD-1 promoter methylation (Ricci et al. 2019), hydroxymethylation (Gambichler et al. 2019), or circulating tumor cells (Riethdorf et al. 2019) are of prognostic relevance in MCC patients. Nevertheless, the data of most studies in this field need to be substantiated by larger prospective investigations.

In the present study, we carefully matched control groups with regard to age and gender. This is of particular importance, since it has been shown that markers of inflammation in peripheral blood increase with age (Fest et al. 2018). However, in our cohort of MCC patients, PIV did not correlate with age (*p* = 0.15), which could be explained by the fact that almost all patients were of similar, old age. Similar to age, gender-dependent differences of CBC-based inflammatory markers have been reported in patients suffering from different cancers (Fest et al. 2018; Li et al. 2018). Consequently, to avoid these confounders, we matched MCC and control groups investigated with respect to both age and gender. In the present study, we could clearly show that the PIV of MCC patients is significantly increased compared to healthy controls further increases depending on stage of the diseases. These data indicate that systemic immune-inflammation responses might not only be useful as a descriptive biomarker when quantified by the PIV, but also hint toward a causal role in tumor development and progression in MCC patients. Said differently, patients with higher MCC stage were also shown to have a higher PIV indicating that systemic immune-inflammation responses increase with tumor size and progression. In line with these observations, a PIV greater than 372 at time of first diagnosis was significantly and independently associated with MCC recurrence, as also supported by a Youden index of 0.58, which is considered a measure of a diagnostic test's ability to balance sensitivity and specificity. Importantly, a Youden index over 0.50 meets empirical benchmarks for being administered as a diagnostic test (Schistermann et al. 2008; Polley and Dignam 2021). However, such an association could not be demonstrated for MCC-specific death. This finding could be explained by the relatively small sample size leading to a low number of events. This limitation may also be the reason for the observation that other expected survival predictors, including immunosuppression and disease stage, were not significantly associated with MCC-specific survival in the present MCC cohort (Becker et al. 2017, 2019).

In conclusion, we determined, for the first time, the prognostic ability of the promising CBC-based biomarker PIV in MCC patients and observed that PIV is increased in MCC patients in dependence on disease stage and independently predicts MCC recurrence. These data strongly support prospective validation of the PIV in a larger sample size.

## Data Availability

The data presented in this study are available on reasonable request from the corresponding author.
